# Duration of mood effects following a Japanese version of the mood induction task

**DOI:** 10.1371/journal.pone.0293871

**Published:** 2024-01-05

**Authors:** Yasunaga Monno, Norberto Eiji Nawa, Noriko Yamagishi

**Affiliations:** 1 Research Organization of Open Innovation and Collaboration, Ritsumeikan University, Ibaraki, Osaka, Japan; 2 Center for Information and Neural Networks, Advanced ICT Research Institute, National Institute of Information and Communications Technology, Suita, Osaka, Japan; 3 Graduate School of Frontier Biosciences, Osaka University, Suita, Osaka, Japan; 4 College of Global Liberal Arts, Ritsumeikan University, Ibaraki, Osaka, Japan; COMSATS University Islamabad, PAKISTAN

## Abstract

Researchers have employed a variety of methodologies to induce positive and negative mood states in study participants to investigate the influence that mood has on psychological, physiological, and cognitive processes both in health and illness. Here, we investigated the effectiveness and the duration of mood effects following the mood induction task (MIT), a protocol that combines mood-inducing sentences, auditory stimuli, and autobiographical memory recall in a cohort of healthy Japanese adult individuals. In Study 1, we translated and augmented the mood-inducing sentences originally proposed by Velten in 1968 and verified that people perceived the translations as being largely congruent with the valence of the original sentences. In Study 2, we developed a Japanese version of the mood induction task (J-MIT) and examined its effectiveness using an online implementation. Results based on data collected immediately after induction showed that the J-MIT was able to modulate the mood in the intended direction. However, mood effects were not observed during the subsequent performance of a cognitive task, the Tower of London task, suggesting that the effects did not persist long enough. Overall, the current results show that mood induction procedures such as the J-MIT can alter the mood of study participants in the short term; however, at the same time, they highlight the need to further examine how mood effects evolve and persist through time to better understand how mood induction protocols can be used to study affective processes more effectively.

## Introduction

Moods and emotions are thought to modulate important functions that are essential for adaptive behavior, such as attention, memory, cognition, and perception [1–4]. Though the link between affective processes and such functions is most clear in individuals suffering from mood disorders (e.g., [5]), studies involving healthy individuals also provide evidence supporting the existence of such connection. For instance, positive mood has been shown to enlarge the scope of visual attention [1], improve response time in a visual search task [6], and impair working memory storage capacity [7]. Furthermore, positive and negative mood have been shown to affect performance in the Tower of London task [8, 9], a task that involves planning and problem-solving.

Laboratory studies investigating the effect of mood on various processes typically employ mood induction protocols to steer the mood of participants toward a target state where changes associated with the induced mood state can be observed. Indeed, a great variety of protocols have been employed in the literature [10, 11]. One of the first systematic protocols was proposed by Velten [12]. In the Velten procedure, participants read a series of short sentences describing relatable everyday life situations and are instructed to try to experience the mood expressed by the sentences. Importantly, the sentences have a positive, negative, or neutral flavor depending on the valence of the mood intended to be induced by the experimenter. There are 60 sentences of each valence in the original protocol [12].

Another commonly used approach to induce mood is to expose experiment participants to music stimuli [13, 14]. Typically, such protocols employ excerpts from classical music pieces or movie soundtracks thought to be congruent with the target mood state (e.g., “happy” pieces in the positive mood condition and “sad” pieces in the negative mood condition [1]). Music appears to be an effective instrument to alter mood, and naturally the variety of music stimuli employed across studies is substantial [10, 11, 15]. Though some musical patterns are shown to be universally recognized as expressing specific emotions [16], cognitive and affective responses elicited by music stimuli are also thought to be influenced by the cultural background of experiment participants [16, 17] and other individual characteristics such as age and previous familiarity with the stimuli genre. This highlights the critical need to thoroughly assess whether and how the music stimuli to be employed in a mood induction protocol has the intended effect in the specific cohort of participants to be tested.

Another mood induction approach involves having participants recall personal memories associated with specific affective states. Memories of personally experienced events, i.e., autobiographical memories, are often associated with affective responses experienced at the time of occurrence of the event and/or resulting from subsequent re-appraisals of the event [18]; such affective responses are likely to be revived during memory recall [19]. In protocols involving autobiographical memory recall, participants are usually asked to prepare a list of memories to be used in the study, so that relevant aspects of the memories can be assessed beforehand [20]. One advantage of this approach is that it capitalizes on the personal nature of autobiographical memories, making it potentially a very powerful and effective way to induce changes in mood.

Robinson and colleagues [21] proposed a generalized mood induction task (MIT) that combines these three different approaches in a single framework. In the MIT, participants are requested to read the 60 Velten sentences of the target mood set (i.e., positive, negative, or neutral) while trying to recall personally experienced events that can be related to the sentences. To further reinforce the induction of the target mood state, participants are exposed to music stimuli congruent with the target mood state throughout the entire process. The effectiveness of the MIT has been verified in the past using self-report data based on visual analog scales (VAS) and other metrics that are less susceptible to demand characteristics, such as the Tower of London (ToL) task [8, 22, 23]. However, despite the importance of understanding the duration of mood effects induced by mood induction protocols, only a few studies have acknowledged or specifically addressed this issue [24, 25].

To bridge this gap, this study assessed mood at multiple times following induction using multiple measures. In tandem with the VAS, we employed a well-established method to assess changes in mood, as employed in [26], i.e., the Positive and Negative Affect Schedule (PANAS) [27], as well as its extended version (PANAS-X) [28]. Because this study involved Japanese participants, all the material had to be presented in Japanese. Surprisingly, we were unable to find a well-assessed and tested Japanese version of the Velten sentences in the literature, even though they have been widely used in psychology research studies [10, 11]. To address this, we translated the sentences to Japanese. In Study 1, we checked whether the valence communicated by the translated sentences matched the intended valence of the original sentences. In this process, we also developed a set of 60 self-referential neutral sentences to align the neutral sentences more closely with the positive and negative sentences. As pointed out in [26], while the positive and negative sentences are all written using a first-person perspective (e.g., “I feel terribly tired…”), the content and the format of the original set of neutral sentences make them much less relatable (e.g., “This book or any part thereof must not be reproduced in any form”). In Study 2, we implemented an online version of the MIT in Japanese (J-MIT) and evaluated its effectiveness in a group of Japanese university students. Subjective ratings (VAS, PANAS, PANAS-X) were collected throughout the procedure to verify whether the J-MIT modulated participants’ mood in the intended manner, and to examine the duration of such effects. Participants also performed the Tower of London task right after induction to verify whether the J-MIT had a significant impact in executive control functions, as previously reported [8].

## Study 1

### Method

#### Participants

Four hundred sixteen individuals (208 identified as women, 208 identified as men, mean age 21.5 years old, *SD* = 1.50, range 20–28 years old) were recruited using the services of an online survey company (Macromill, Inc., Tokyo, Japan) in September 2020. Participants were compensated with proprietary points that could be later exchanged for gifts. Data were anonymized before analysis. The study was approved by the local research ethics committee of the National Institute of Information and Communications.

#### Materials

Participants evaluated four groups of Japanese sentences: Velten-Positive, Velten-Negative, Velten-Neutral, and Self-Neutral (for the full list, see S1 File). Velten-Positive, Velten-Negative, and Velten-Neutral were the Japanese translations of the original Velten sentences. Sentences were translated to Japanese by professional translators (Ability InterBusiness Solutions, Inc., Hiroshima, Japan). Authors fluent in both English and Japanese verified that the translations retained the meanings conveyed by the original sentences.

One critical issue concerning the original Velten neutral sentences, as pointed out in [26], is that the neutral sentences are not self-referential: unlike the positive and negative sentences, it is much harder to relate to the contents described by the originally neutral sentences (e.g., "The orient express travels between Paris and Istanbul”). The primary goal of a neutral mood condition is to serve as a comparison baseline, by being as emotionally insipid as possible. In practice, a neutral condition aims primarily to exert minimal influence in the mood of participants while being as similar as possible to the other conditions. To enhance similarity, we developed a new set of 60 neutral sentences (hereinafter, the Self-Neutral set) by combining 20 neutral statements with a self-referential component previously employed in [26] with a group of newly created 40 sentences for the purposes of this study (see S1 Text and S2 File for details). Participants evaluated the self-referential neutral statements along with the translations of the original Velten sentences.

#### Procedure

Participants were divided into four groups to reduce participant load and promote better response quality (Table 1). Each group rated a non-overlapping set of 60 sentences out of the 240 sentences. Each set had an equal number of sentences from each one of the 4 valence types (Velten-Positive, Velten-Negative, Velten-Neutral, and Self-Neutral). We chose this scheme to minimize the chances that mood states inadvertently induced during the rating of the sentences influenced the participants’ evaluations (for example, if a group of participants were asked to evaluate the entire set of 60 Velten-Negative sentences). Participants were asked to rate how “happy” they would feel if experiencing the situations described in the sentences, using a 9-point scale ranging from “extremely unhappy” (1) to “extremely happy” (9). The order of the sentences was randomized across participants. We used R (ver. 4.0.3) to analyze the results.

**Table 1:** Number of participants and mean age (standard deviation) for each group in Studies 1 and 2.

Study	Participant group	Total	Women	Men	Mean age (SD)	Age range
**Study 1**	G1	104	52	52	21.18 (1.44)	[20–28]
	G2	104	52	52	21.72 (1.60)	[20–27]
	G3	104	52	52	21.53 (1.50)	[20–25]
	G4	104	52	52	21.44 (1.43)	[20–27]
**Study 2**	Positive	25	13	12	21.96 (1.51)	[20–26]
	Negative	28	14	14	21.86 (1.60)	[20–25]
	Neutral	25	12	13	22.36 (1.58)	[20–25]
	Neutral-Self	26	12	14	22.04 (1.59)	[20–27]
	Soundscape-Neutral	30	12	18	21.40 (1.25)	[20–24]

No significant gender differences across conditions were detected (Chi-squared test, *p* = .995). No significant age differences were also found for Study 1 (*p* = .074) and Study 2 (*p* = .212) across conditions.

### Results

The mean ratings differed significantly across sentences of different valence. Table 2 shows the mean ratings given by each group of participants (G1 to G4) to the sentences of different valences. To determine whether there were effects of valence and/or participant group, we conducted a mixed-design two-way ANOVA with valence (Velten-Positive, Velten-Negative, Velten-Neutral, and Self-Neutral) as the within-subjects factor and participant group (G1 to G4) as the between-subjects factor. Mendoza’s test indicated that the assumption of sphericity was violated (*χ*^2^(23) = 755.17, *p* < .001), thus, the Greenhouse-Geisser correction was used (*ε* = 0.48). Both main effects of valence (*F*(1.44, 595.15) = 844.91, *p* < .001, *η*_*p*_^*2*^ = .67) and participant group (*F*(3, 412) = 8.99, *p* < .001, *η*_*p*_^*2*^ = .06) were found to be statistically significant. In addition, the interaction between both factors was also found to be significant (*F*(4.33, 595.15) = 5.58, *p <* .001, *η*_*p*_^*2*^ = .04).

**Table 2:** Mean ratings of “happiness” for the four sentence types across the four groups.

Group	Velten-Positive	Velten-Negative	Velten-Neutral	Self-Neutral
**G1**	6.08 (1.09)	3.83 (1.00)	4.97 (0.55)	5.52 (0.84)
**G2**	6.28 (1.23)	3.52 (1.01)	4.81 (0.57)	5.27 (0.78)
**G3**	6.77 (1.06)	3.64 (0.89)	5.05 (0.61)	5.79 (0.77)
**G4**	6.70 (1.11)	3.56 (0.96)	5.18 (0.53)	5.49 (0.68)

Standard deviations are in brackets.

The simple main effect of the valence was tested for each participant group. Mendoza’s test indicated that the assumption of sphericity was violated in all levels of valence and thus Greenhouse-Geisser correction was used (G1: *χ*^2^(5) = 174.68, *p* < .001, *ε* = 0.49; G2: *χ*^2^(5) = 203.87, *p* < .001, *ε* = 0.46; G3: *χ*^2^(5) = 148.04, *p* < .001, *ε* = 0.52; G4: *χ*^2^(5) = 223.80, *p* < .001, *ε* = 0.45). The simple main effects of valence were significant in all rating groups (G1: *F*(1.46, 150.56) = 131.72, *p* < .001, *η*_*p*_^*2*^ = .56; G2: *F*(1.39, 142.70) = 194.29, *p* < .001, *η*_*p*_^*2*^ = .65; G3: *F*(1.55, 159.83) = 306.02, *p* < .001, *η*_*p*_^*2*^ = .75; G4: *F*(1.34, 138.11) = 246.05, *p* < .001, *η*_*p*_^*2*^ = .70). Moreover, pairwise comparisons with Bonferroni correction indicated that, for all groups, differences between sentences of different valence types were significant (*p* < .05 corrected).

### Discussion

Results from Study 1 established that the scores were consistent with the mood intended to be induced by the sentences of each valence type. As expected, “happiness” ratings were highest for the Velten-Positive sentences, followed by Self-Neutral, Velten-Neutral, and Velten-Negative. These results indicate that the Japanese translations of the Velten sentences, including the newly developed set of 60 Self-Neutral sentences, appropriately conveyed the intended valence, making them suitable to be used in mood induction protocols.

Interestingly, there was a significant main effect of rating group, as well as an interaction between rating group and valence. Participants were randomly assigned to each one of the 4 groups, making it highly unlikely that the groups had different biases towards the sentences. The most plausible explanation would be that the 4 blocks of sentences accidentally contained small differences regarding the construct being evaluated (“How happy would you be in this situation?”), that ended up being reflected in the ratings given by the participants. Most importantly, while there was a significant main effect of rating group, results from simple main effects tests of valence within each group showed that the Velten-Positive sentences were consistently judged to be more happiness-evoking than the other sentences, followed by the Self-Neutral sentences, the Velten-Neutral sentences, and last the Velten-Negative sentences. As expected, the latter group of sentences had the lowest happiness-evoking ratings across all groups. This shows that despite the differences across groups, all participants perceived the Velten-Positive sentences as being the most positive sentences, and the neutral sentences (both Self-Neutral and Velten-Neutral) as being more positive than the Velten-Negative sentences. Importantly, the differences observed here do not pose a consequential problem because during mood induction, participants are only exposed to sentences of the same valence type.

## Study 2

Having established that the perceived valence of the translated sentences matched the valence intended to be conveyed by the original sentences, and that the valence of the newly developed Self-Neutral sentences was in the appropriate range relative to the Velten-Positive and Velten-Negative sets, we performed an online study to assess the duration of the mood induction effects, and the overall effectiveness of the J-MIT in Japanese healthy individuals. Self-report ratings were collected at key timepoints during the experiment; in addition, participants undertook the Tower of London (ToL) task right after the completion of the mood induction to verify potential effects of mood on processes associated with executive control functions [9]. Robinson et al. [8] employed the one-touch ToL task (see Methods below) following the mood induction task and showed that, compared to participants in the neutral condition, participants in the positive and negative conditions displayed an increase in the number of errors as the task difficulty increased. We expected to observe a similar performance degradation (i.e., higher error rates) among Study 2 participants assigned to the positive and negative conditions compared to those in the neutral conditions.

### Method

#### Data collection periods

Data in Study 2 was collected at two different occasions (September 2020 and February 2021). In the first occasion, data were collected using a between-subjects design with 4 experimental conditions: positive (Velten-Positive sentences paired with a positive music stimuli), negative (Velten-negative sentences paired with a negative music stimuli) and two neutral conditions (Velten-neutral sentences or Self-Neutral sentences paired with a neutral music stimulus) (Details described in Table 3). However, results from the first data collection indicated that the music stimulus used in the two neutral conditions [21] appeared to have had an unintended negative mood inducing effect in our participants (more details in Results). To address that problem, we collected additional data where participants listened to audio clips of environmental sounds (“soundscapes”) instead of the music stimulus employed previously. This new condition was named “Soundscape-Neutral” (Table 3). Even though the second data collection was performed at a different occasion, all other aspects of the experimental protocol were identical to the first data collection. We also confirmed that all participants in the second data collection were taking part in this study for the first time.

**Table 3:** Sentence, audio, and text/background color types for the five J-MIT conditions.

MIT condition	Sentences	Music or soundscape	Text color (RGB color)	Background (RGB color)
**Positive**	Velten-Positive	Piano Concerto No. 4, Op. 58 in G Major: III. Rondo: Vivace	Orange (#FF8000)	Yellow (#FFFF00)
		Serenade No. 13 KV 525 G-Major: I. Serenade. Llegro		
**Negative**	Velten-Negative	Adagio for strings, Op. 11 by Samuel Barber	Gray (#808080)	Blue (#0000FF)
		Adagio in G Minor		
**Neutral**	Velten-Neutral	The Planets, Op. 32: VII. Neptune, the Mystic	Black (#000000)	White (#F6F6F6)
**Self-Neutral**	Self-Neutral	The Planets, Op. 32: VII. Neptune, the Mystic	Black (#000000)	White (#F6F6F6)
**Soundscape-Neutral**	Self-Neutral	Soundscape 1: School-Street Ambience	Black (#000000)	White (#F6F6F6)
		Soundscape 2: Night Crickets Back Porch		

Note that in the Positive, Negative, and Soundscape-Neutral conditions, but not Neutral and Self-Neutral conditions, participants could choose the auditory stimulus to be used during the task from two options.

#### Participants

One hundred forty-one university students (66 identified as women, 75 identified as men, mean age 21.9 years old, *SD* = 1.53, ranged 20–27 years old) took part in the first data collection. A separate group of 43 students took part in the second data collection (17 identified as women, 26 identified as men, mean age 21.4 years old, *SD* = 1.27, ranged 20–24 years old). Participants were recruited via a social media app (Twitter) using an account maintained by a volunteer of the local university community solely for the purpose of recruiting study participants. A call was sent to the approximately 5,000 followers of that account in September 2020 and February 2021; interested users were redirected to an online form, which described details of the procedures involved in the study and the participation requirements, namely, to be at least 20 years old (the age of legal majority in Japan at the time of the study), to be currently enrolled as an undergraduate or graduate student in an university and to have internet access. Participants were told that they would receive 3,000 yen upon completion of the experiment. People who declared to meet all requirements were asked to provide their contact information, name, and gender. All individuals were then contacted via email; in accordance with the principles stated in the Declaration of Helsinki, those who wished to voluntarily sign up for the study were requested to fill out and submit an online consent form. In addition, participants were asked to complete the paperwork necessary to receive the monetary compensation. The authors could only identify participants by looking up a table that was stored on a computer that was kept offline. The study was approved by the local research ethics committee of the National Institute of Information and Communications.

All participants were requested to complete the Center for Epidemiologic Studies Depression Scale questionnaire (CES-D) [29] and the Social Desirability Scale-17 (SDS-17) [30]. In the first data collection, participants whose CES-D scores were equal or greater than 16 were not assigned to the Negative condition; all other participants were randomly assigned to either the Positive, Negative, Neutral or Self-Neutral conditions. In the second data collection, all participants were assigned to the Soundscape-Neutral condition regardless of CES-D score; however, those who had a score equal or greater than 16 were excluded from the analysis [29]. The characteristics of the participants utilized in the analysis for each condition are presented in Table 1.

The website used in the study was hosted on a proprietary cloud server. Participants were issued individual accounts and passwords and accessed the website using a browser of their choice, from their own computers. They were free to access the site at any time within a specified time window (from 6 AM to 12 PM). Timestamps were collected (e.g., login time, study completion, etc.) as participants proceeded with the tasks. The progress of each participant was monitored to prevent them from performing the steps involved in the study more than once.

#### Materials

The text and audio stimuli pairs employed in each one of the 5 conditions are shown in Table 3. The Positive, Negative, and Neutral conditions followed the specifications described in [21]. The Self-Neutral condition employed the Self-Neutral sentences developed in Study 1 with the music stimulus used in the Neutral condition. In the Soundscape-Neutral condition, the Self-Neutral sentences were used with one of two soundscape audio clips (in place of the Neutral music stimulus). We selected the soundscapes by referring to a publicly available database of valence-arousal-scored audio clips [31]. While this database evaluated the valence and arousal of 6-second excerpts of the soundscapes, in this study we employed the full-length recording from where the clip was extracted, after verifying that they largely retained the audio characteristics of the 6-second excerpts. Soundscapes were processed to minimize microphone static noise and abrupt changes in loudness (e.g., caused by a car passing nearby in one of the clips). Finally, a fade-in/fade-out filter was used at the start and end of the audio files so that they could be continuously played on a loop. The original sources of Soundscape 1 and Soundscape 2 (Table 3) were obtained from https://freesound.org/people/15050_Francois/sounds/325806/ and https://freesound.org/people/hdfreema/sounds/333221/ each.

The background and text colors in both the positive and negative conditions are in line with findings from research that investigated the interplay between colors and emotions [32, 33]. Specifically, music that is generally perceived as happier (major key) is often associated with colors that are brighter, more saturated, and more yellowish, while music that is generally perceived as more sad (minor key) is often associated with colors with the opposite characteristics, i.e., leaning towards bluish hues [32]. Largely consistent with such findings, people show increased propensity to depict their mood with the color yellow following an induction of a joyous mood [33]. Based on these results, the positive mood condition used orange text on a yellow background, while the negative condition had gray text on a blue background (Table 3).

#### Mood induction task procedure

The mood induction task followed the procedure outlined in [21]. In the Positive, Negative, and Soundscape-Neutral conditions, at the beginning of the task, participants were instructed to listen to two audio samples and choose the one that made them feel more like in the target mood (i.e., positive, negative, or neutral). Participants had to listen to each one of the clips for at least one minute, and they were only allowed to make a choice after listening to both samples. They were free to listen for longer if they wished.

Following the auditory stimulus selection, task instructions were displayed using black (RGB: #000000) text on a white screen (RGB: #FFFFFF). After the instruction, the task started after a button-press, when the colors of the text and background gradually changed to the colors of the target mood.

During mood induction, sentences from the assigned condition were displayed on the screen, one by one, in the same order, for all participants. Participants were instructed to associate the contents of the text with autobiographical memories of personally experienced events. Each sentence was presented for at least 12 seconds; after that time had elapsed, participants could proceed to the next sentence by clicking a button right under the text. The selected auditory stimulus was played throughout during the presentation of the sentences. After the entire set of 60 sentences had been displayed, a surprise test was conducted to check participant attention. Participants were asked to choose the 2 sentences out of 4 that had not been used during induction. Targets were the last sentence (sentence 60) and the one before that (sentence 59). Two participants (one in the Neutral condition and another in the in Soundscape-Neutral condition) failed to select at least one of the target sentences and were excluded from the analyses.

#### Tower of London (ToL) task

**Fig 1:**
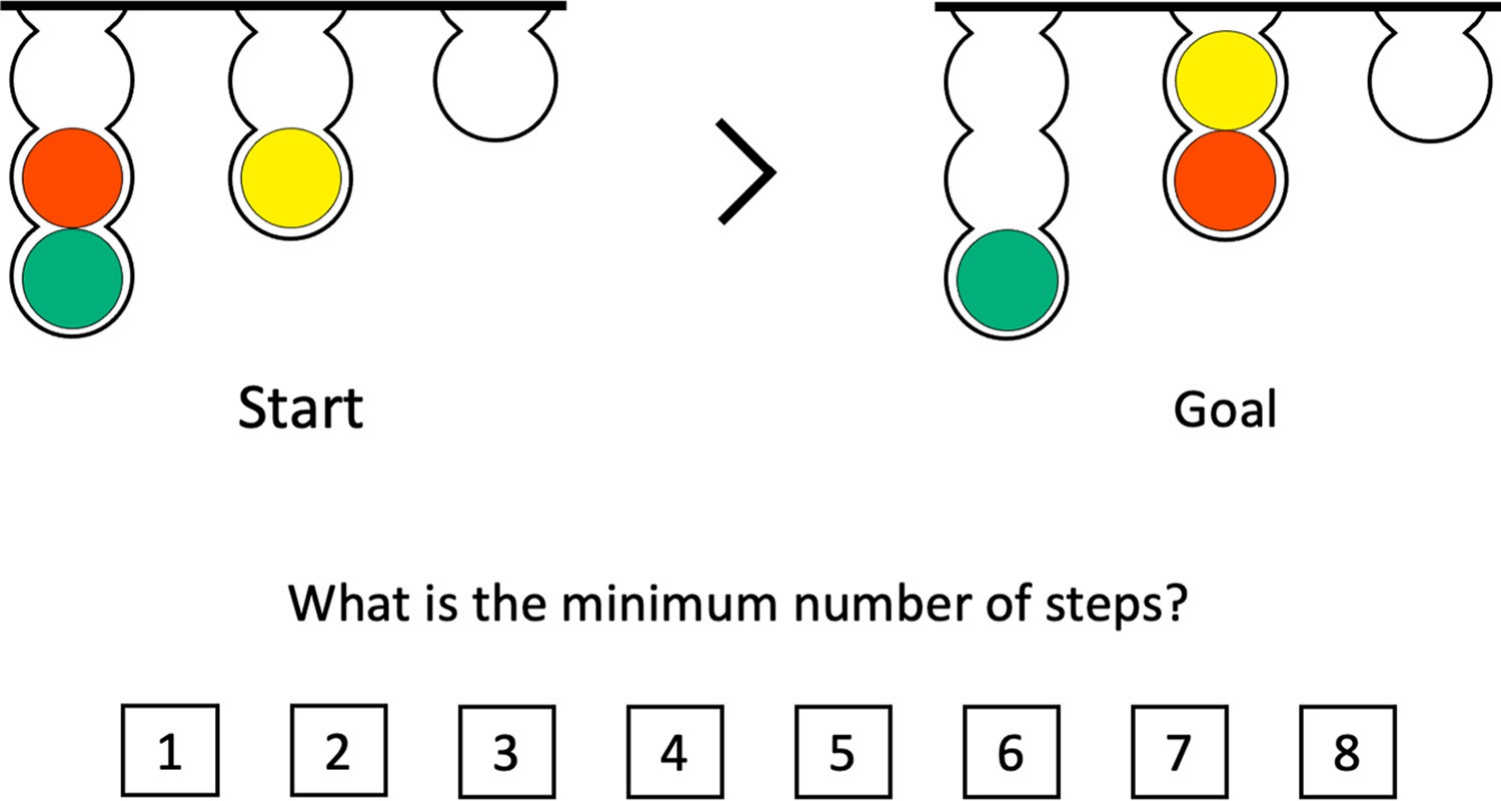
Example of a Tower of London task trial. The start and goal configurations were presented on the left and right respectively. Participants had to mentally calculate the minimum number of steps necessary to transfer the balls from the start configuration to the goal configuration. They were asked to respond by clicking the button displaying the correct number of moves (in the example, the correct answer is 3). The trial only ended when the participant clicked the button displaying the correct answer.

The ToL is based on the classic problem of the Tower of Hanoi [22]; there are three billiard balls of different colors, which can be distributed along three pockets of different depths (1, 2, and 3). The task of participants is to move the balls to form the goal configuration (Fig 1). Balls can only be moved one at a time, and spots available in the other pockets can be used as temporary holders. Only the top ball of each pocket can be moved, and balls cannot be stacked higher than the depth of the pockets. In the one-touch ToL employed in this study, participants are required to mentally rehearse the movements and calculate the minimum number of steps to achieve the goal configuration, without actually manipulating the balls. Participants are requested to press the button on the screen displaying their answer (i.e., number of steps) after reaching a decision. If the answer is correct participants are allowed to move to the next trial; however, if the answer is incorrect, participants are requested to try again until the correct answer is provided. As such, the average number of incorrect button-presses across trials provides a measure of task performance. Task difficulty increases as the minimum number of the steps to arrive to the goal configuration becomes larger. The ToL is known to probe processes associated with executive control and working memory that are typically recruited during planning and problem-solving. The ToL has been used to examine the extension to which such capacities are affected in lesion studies [34] and other neurological disorders, such as Parkinson’s disease [35, 36].

Before performing the ToL, participants watched an instruction video (recorded for the purposes of this study) explaining the rules of the task. Participants could watch the video as many times as necessary. At the start of the ToL, participants were given two practice trials to verify their comprehension. At every trial, a warning was displayed if the answer was incorrect; participants were prompted to continue trying until they provided the correct answer. The next trial started one second after a correct answer was provided. There were two trials for each difficulty level from 1 to 3, and four trials for each difficulty level from 4 to 6, totaling 18 trials. The order of the trials was randomized across participants. The practice trials were of difficulty levels 2 and 3. The colors of the balls were chosen using the color universal design (CUD) guidelines [37].

#### Washout

To minimize the possibility that changes in mood induced by the protocol outlasted the duration of the study, participants had to take part in a “washout” (WO) session right after the completion of all tasks. The session consisted of watching movie clips of “cats”, “waterfalls” or “marine scenes.” Participants selected the movie clip they wished to watch by clicking the corresponding button. All participants were requested to watch the movie clip of their preference for at least 5 minutes, regardless of the experimental condition. Movie clips were obtained from the NHK Creative Library (https://www.nhk.or.jp/archives/creative/).

#### Mood ratings

Participants evaluated their mood during the experiment using two methods. Levels of momentary happiness and sadness were assessed by means of visual analogue scales (VAS) as in [38]. We also collected ratings of depression and boredom using VAS. Data were collected in the range of 0 (“Not at all”) to 100 (“Extremely”) using whole numbers. Because we wanted participants to make the evaluations in an intuitive manner, the number corresponding to the position of the marker on the VAS slider was not displayed.

In the PANAS and PANAS-X, individuals evaluate how well different affect related labels (e.g., “active”) describe their mood state using a 6-point Likert scale (1: not at all; 6: extremely) [39]. We computed the mean levels of “positive affect” (10 labels) and “negative affect” (10 labels) using the 20 labels in the PANAS. We further computed scores of “joviality” (8 labels) and “sadness” (5 labels) using the labels in the PANAS-X. The order in which labels were presented was randomized once and remained fixed throughout the entire study and across participants.

**Fig 2:**
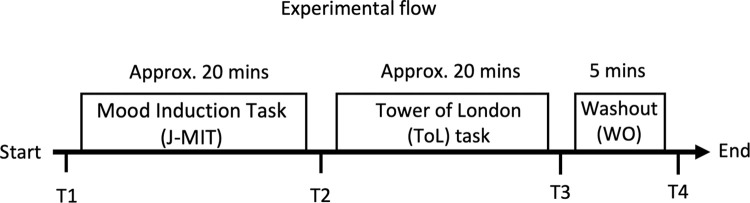
Timeline of Study 2. Participants were randomly assigned to one of the conditions and underwent the mood induction task (J-MIT). Next, participants performed the Tower of London task (ToL). Finally, to ensure that the induced moods did not persist after the experiment, participants took part in a “washout” (WO) session. Mood ratings were taken at four distinct time points (T1 to T4): before induction (T1), after induction (T2), after performing the Tower of London Task (T3), and after the “washout” session (T4).

Mood data were collected four times during the experiment: at the start of the experiment (T1), right after the mood induction (T2), right after the ToL task (T3), and right after the washout session (T4) (see Fig 2 for a schematic of the experiment timeline). A surprise test was given right after the first measurement (T1) to check the attention of participants: 2 labels that do not belong to the PANAS-X (“sleepy” and “sense of guilt”) appeared alongside two other legitimate PANAS-X labels and participants were prompted to select the two labels that did not appear in the list. Two participants failed to identify at least one of the decoys and were excluded from the analyses (one participant in the Positive condition and one in the Self-Neutral condition).

The difference between the ratings given at T2 and T1 (T2-T1) reflect immediate mood effects induced by the J-MIT, while the difference between ratings given at T3 and T1 (T3-T1) reflect sustained mood effects, i.e., effects that survive the performance of the ToL. Data were analyzed using a two-way mixed-design ANOVA with induced mood as between subject factor (Positive, Negative, Neutral, Self-Neutral and Soundscape-Neutral) and time as within subject factor (T1, T2 and T3). Note that mood ratings at T4 were only used to check whether the mood of participants was not substantially negative at the end of the experiment relative to baseline.

In addition to these analyses, we explored the potential influence of gender on the effects yielded by the J-MIT. Gender may influence subjective emotional responses [40], for example, women have been reported to exaggerate negative emotions more than men [41]. To examine if that was the case here, we added gender as a factor and conducted an additional three-way mixed-design ANOVA based on the PANAS Positive and PANAS Negative scores.

### Results

#### Mood ratings

**Fig 3:**
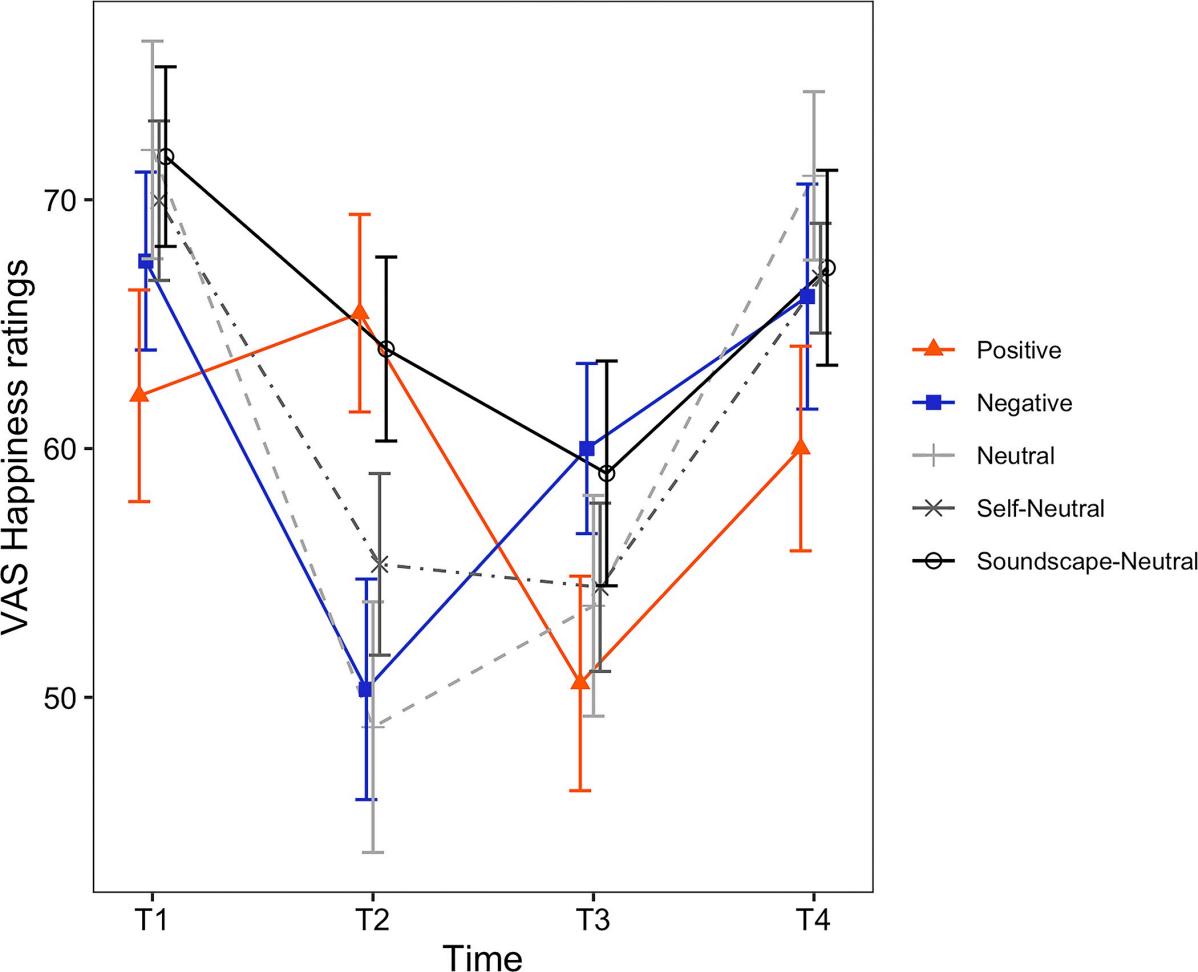
Timecourses of the VAS Happiness ratings. The horizontal axis shows the time of measurement. Each line depicts a different condition. Error bars represent the standard error of the mean (SEM).

**Fig 4:**
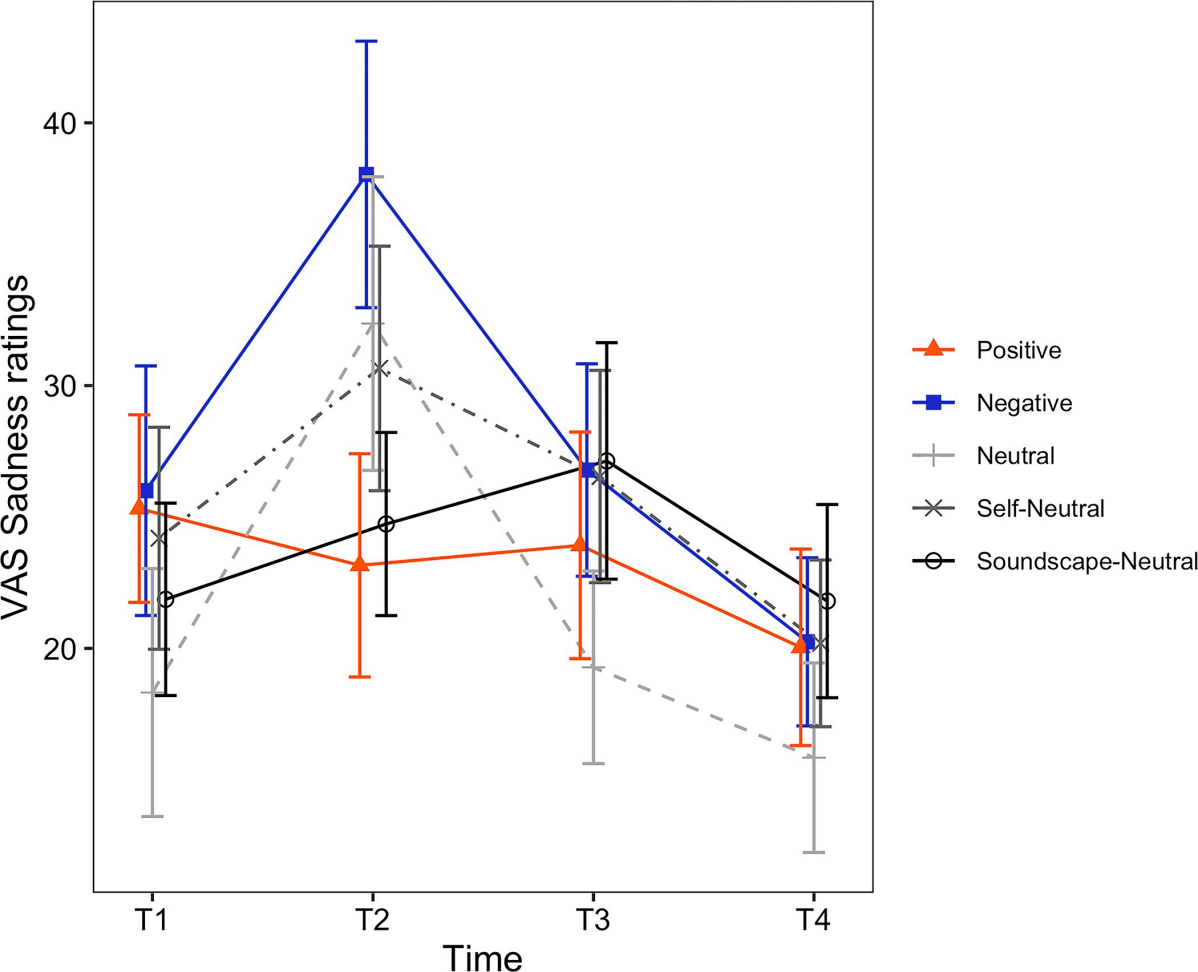
Timecourses of the VAS Sadness ratings. The horizontal axis shows the time of measurement. Each line depicts a different condition. Error bars represent the standard error of the mean (SEM).

**Fig 5:**
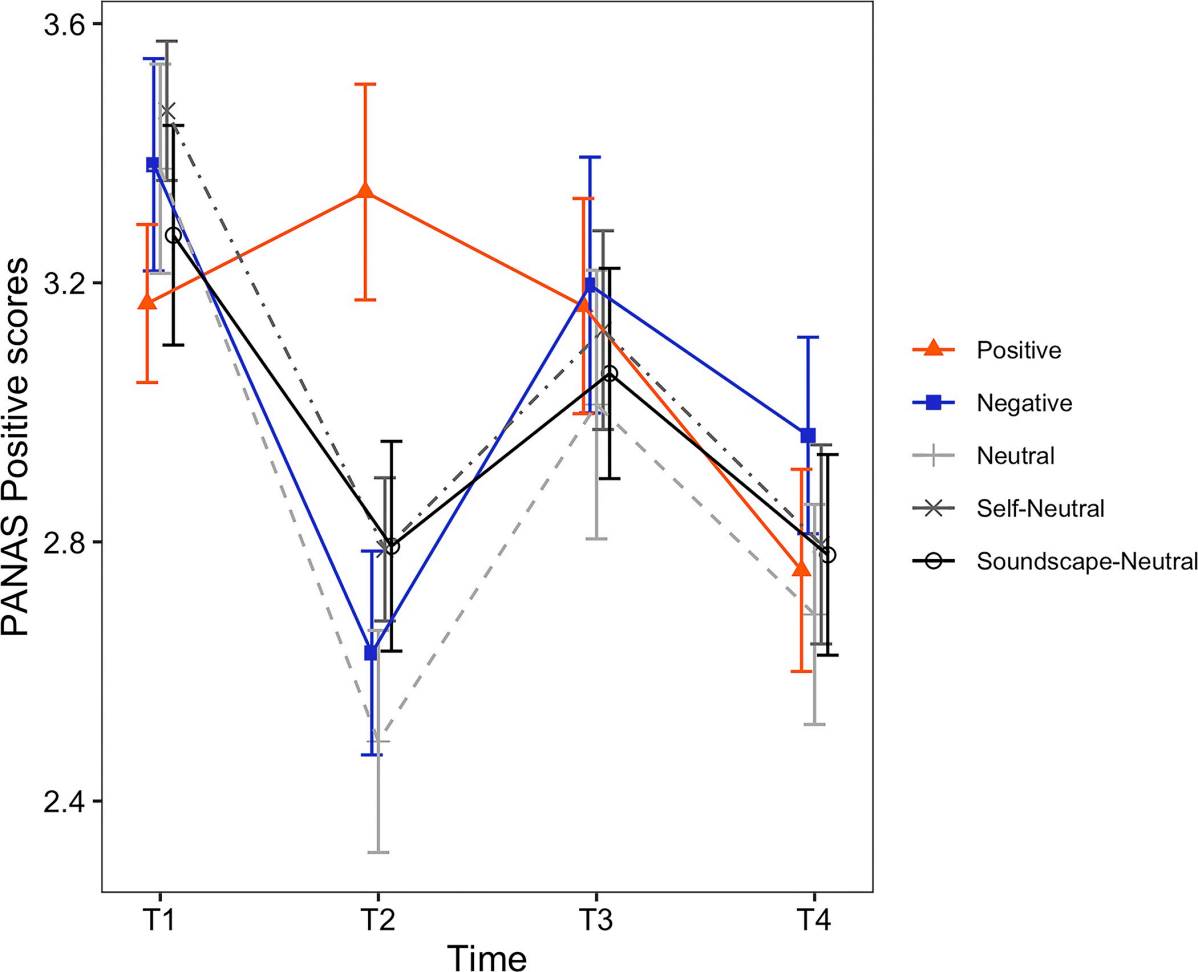
Timecourses of the PANAS Positive scores. The horizontal axis shows the time of measurement. Each line depicts a different condition. Error bars represent the standard error of the mean (SEM).

**Fig 6:**
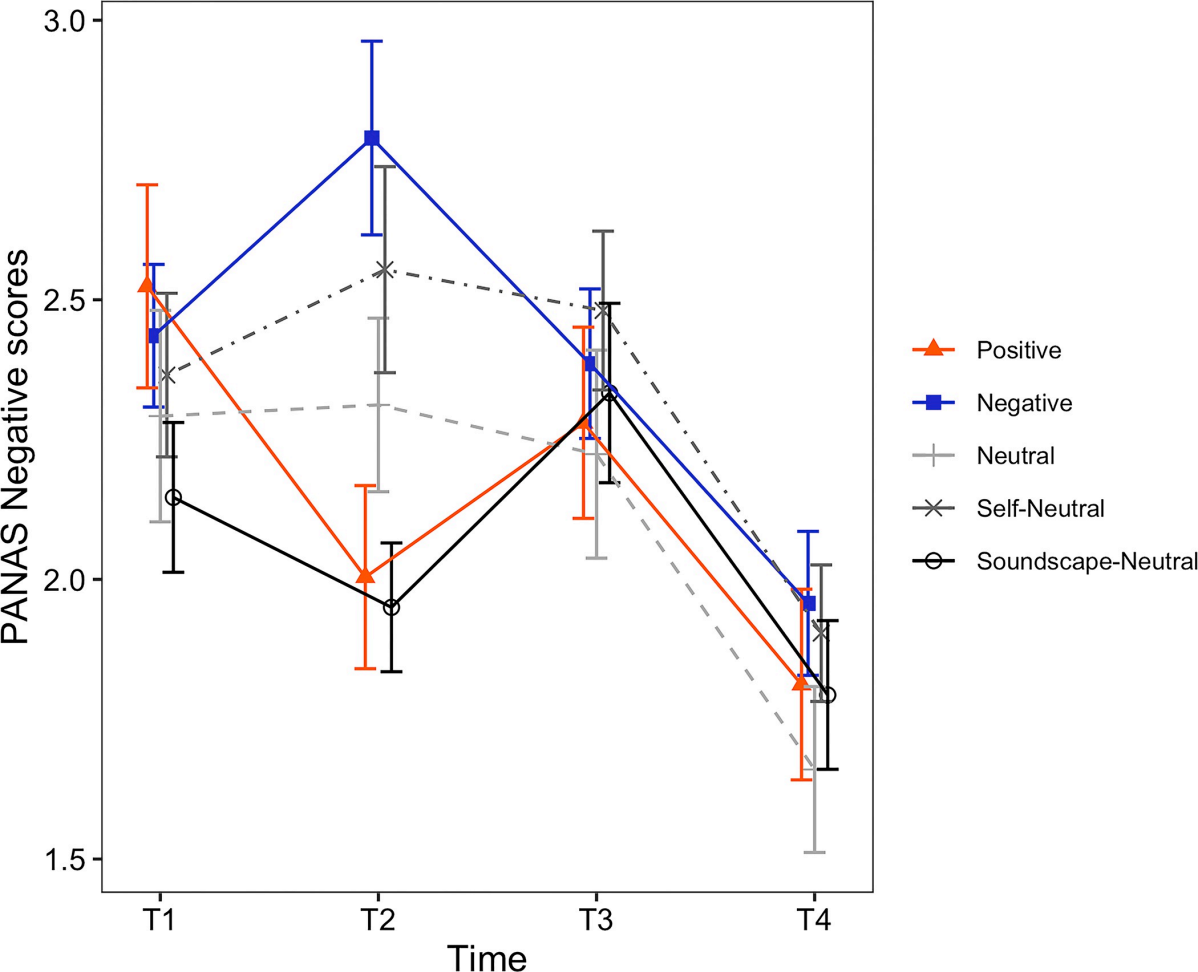
Timecourses of the PANAS Negative scores. The horizontal axis shows the time of measurement. Each line depicts a different condition. Error bars represent the standard error of the mean (SEM).

**Fig 7:**
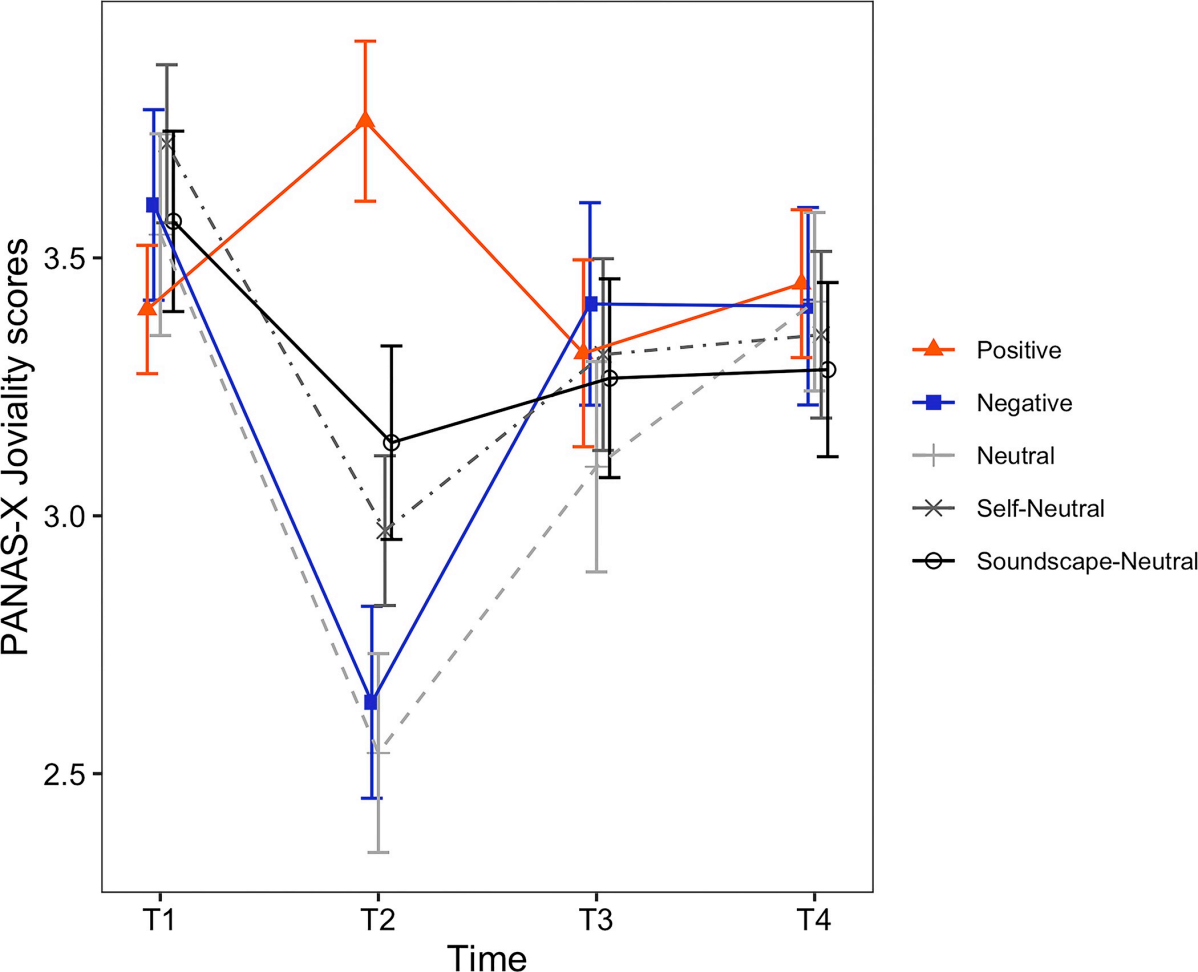
Timecourses of the PANAS-X Joviality scores. The horizontal axis shows the time of measurement. Each line depicts a different condition. Error bars represent the standard error of the mean (SEM).

**Fig 8:**
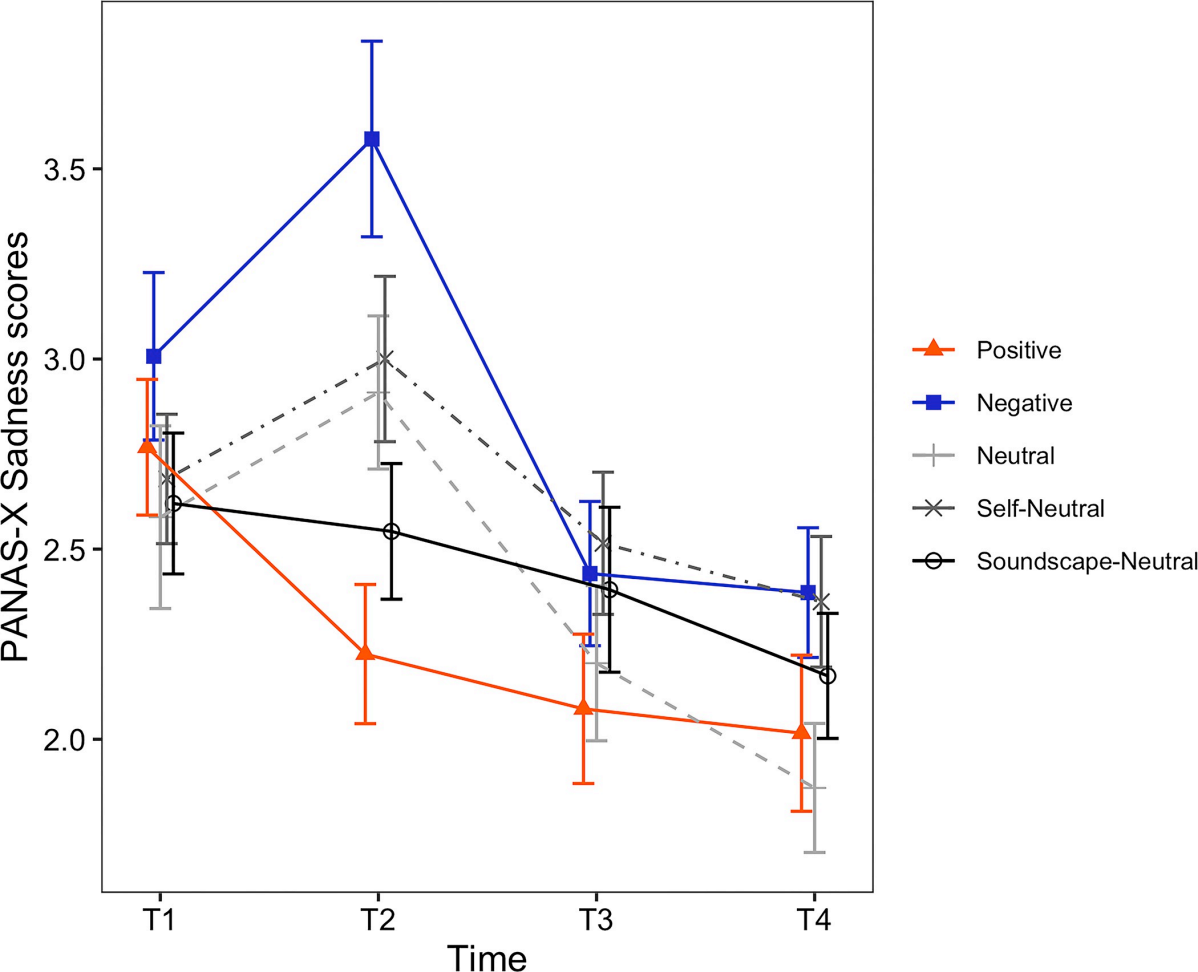
Timecourses of the PANAS-X Sadness scores. The horizontal axis shows the time of measurement. Each line depicts a different condition. Error bars represent the standard error of the mean (SEM).

The timecourses of the mood ratings are shown in Figs 3–8. A mixed-design ANOVA was conducted using the various measurements of positive and negative mood, i.e., Happiness and Sadness using VAS, Positive affect and Negative affect using PANAS and Joviality and Sadness using PANAS-X. Statistical results are shown for each one of the scales. Mendoza’s sphericity test was employed and whenever the assumption of sphericity was violated, the Greenhouse-Geiser correction was applied. Multiple comparisons in post-hoc tests were corrected using Shaffer’s modified Bonferroni correction [42]. Statistical significance threshold was set at the *p* < .05 level unless otherwise stated.

*VAS rating scores*. For the VAS Happiness ratings (Fig 3), the main effect of induced mood was not statistically significant (*F*(4, 129) = 0.67, *p* = .617). However, the main effect of time (*F*(1.90, 244.77) = 35.85, *p* < .001, *η*_*p*_^*2*^ = .217) and the interaction between induced mood and time were significant (*F*(7.59, 244.77) = 4.60, *p* < .001, *η*_*p*_^*2*^ = .125). Because there was a significant interaction, we examined the simple main effect of time for each one of the induced moods. The simple main effect of time was significant for all induced mood: Positive (*F*(2, 48) = 11.04, *p* < .001, *η*_*p*_^*2*^ = .315), Negative (*F*(1.63, 44.10) = 13.28, *p* < .001, *η*_*p*_^*2*^ = .330), Neutral (*F*(2, 48) = 13.37, *p* < .001, *η*_*p*_^*2*^ = .358), Self-Neutral (*F*(2, 50) = 10.38, *p* < .001, *η*_*p*_^*2*^ = .293) and Soundscape-Neutral conditions (*F*(2, 58) = 5.78, *p* = .005, *η*_*p*_^*2*^ = .166). Results for the pairwise comparisons of VAS Happiness are shown in Table 4 (Happiness). Values in Tables 4 and 5 are displayed in bold when the difference between the respective timepoints was found to be statistically significant.

**Table 4:** Changes in VAS Happiness and VAS Sadness between different timepoints.

Condition	Scale	T2—T1				T3—T1				T3—T2			
Positive	Happiness	3.32	(16.73)			**-11.56**	**(18.65)**	**➘**	**0.62**	**-14.88**	**(14.19)**	**➘**	**1.05**
	Sadness												
Negative	Happiness	**-17.21**	**(21.45)**	**➘**	**0.80**	**-7.54**	**(14.71)**	**➘**	**0.51**	**9.68**	**(16.29)**	**➚**	**0.59**
	Sadness	**12.04**	**(23.93)**	**➚**	**0.50**	0.79	(17.31)			**-11.25**	**(20.77)**	**➘**	**0.54**
Neutral	Happiness	**-23.20**	**(25.49)**	**➘**	**0.91**	**-18.32**	**(26.00)**	**➘**	**0.70**	4.88	(18.78)		
	Sadness	**14.04**	**(28.04)**	**➚**	**0.50**	0.96	(22.84)			**-13.08**	**(18.73)**	**➘**	**0.70**
Self-Neutral	Happiness	**-14.62**	**(19.54)**	**➘**	**0.75**	**-15.54**	**(22.05)**	**➘**	**0.70**	-0.92	(16.55)		
	Sadness												
Soundscape-Neutral	Happiness	**-7.73**	**(16.53)**	**➘**	**0.47**	**-12.73**	**(24.24)**	**➘**	**0.53**	-5.00	(20.53)		
	Sadness												

Values in bold indicate differences that were statistically significant at the *p* < .05 level. Standard deviations are inside brackets. Arrows indicate whether the value decreased (downward arrow) or increased (upward arrow) significantly between the respective timepoints. The numbers to the right of the arrows represent Cohen’s d values. Empty rows indicate the conditions where the simple main effect of time was not significant, therefore, pairwise comparisons were not performed (i.e., Positive, Self-Neutral and Soundscape-Neutral).

**Table 5:** Changes in PANAS and PANAS-X ratings between different timepoints.

Condition	Scale	T2—T1				T3—T1				T3—T2			
Positive	Positive												
	Negative	**-0.52**	**(0.50)**	**➘**	**1.04**	-0.24	(0.78)			**0.28**	**(0.60)**	**➚**	**0.46**
	Joviality	**0.37**	**(0.71)**	**➚**	**0.52**	-0.09	(0.86)			**-0.45**	**(0.94)**	**➘**	**0.48**
	Sadness	**-0.54**	**(0.64)**	**➘**	**0.85**	**-0.69**	**(0.73)**	**➘**	**0.94**	-0.14	(0.42)		
Negative	Positive	**-0.75**	**(0.78)**	**➘**	**0.97**	-0.19	(0.76)			**0.57**	**(0.92)**	**➚**	**0.61**
	Negative	**0.35**	**(0.89)**	**➚**	**0.40**	-0.05	(0.56)			**-0.40**	**(0.62)**	**➘**	**0.66**
	Joviality	**-0.96**	**(0.97)**	**➘**	**1.00**	-0.19	(0.69)			**0.77**	**(0.96)**	**➚**	**0.81**
	Sadness	**0.57**	**(1.12)**	**➚**	**0.51**	**-0.57**	**(0.85)**	**➘**	**0.68**	**-1.14**	**(1.11)**	**➘**	**1.03**
Neutral	Positive	**-0.88**	**(0.84)**	**➘**	**1.05**	**-0.36**	**(0.87)**	**➘**	**0.42**	**0.52**	**(0.98)**	**➚**	**0.53**
	Negative												
	Joviality	**-1.01**	**(0.87)**	**➘**	**1.15**	**-0.45**	**(1.06)**	**➘**	**0.42**	**0.56**	**(0.82)**	**➚**	**0.68**
	Sadness	0.33	(0.96)			-0.38	(1.13)			**-0.71**	**(0.92)**	**➘**	**0.77**
Self-Neutral	Positive	**-0.68**	**(0.68)**	**➘**	**1.00**	-0.34	(0.94)			**0.34**	**(0.74)**	**➚**	**0.46**
	Negative												
	Joviality	**-0.75**	**(0.89)**	**➘**	**0.84**	-0.41	(1.19)			0.34	(0.98)		
	Sadness	0.32	(0.88)			-0.17	(0.68)			**-0.48**	**(0.62)**	**➘**	**0.78**
Soundscape-Neutral	Positive	**-0.48**	**(0.55)**	**➘**	**0.87**	-0.21	(0.89)			0.27	(0.81)		
	Negative	**-0.20**	**(0.44)**	**➘**	**0.45**	0.19	(0.62)			**0.38**	**(0.70)**	**➚**	**0.55**
	Joviality	**-0.43**	**(0.61)**	**➘**	**0.70**	-0.30	(1.03)			0.13	(0.84)		
	Sadness												

Values in bold indicate differences that were statistically significant at the *p* < .05 level. Standard deviations are inside brackets. Arrows indicate whether the value decreased (downward arrow) or increased (upward arrow) significantly in time between the respective timepoints. The numbers to the right of the arrows represent Cohen’s d values. Empty rows indicate the conditions where the simple main effect of time was not significant, therefore, pairwise comparisons were not performed (i.e., Positive, Neutral, Self-Neutral and Soundscape-Neutral).

For the VAS Sadness ratings (Fig 4), the main effect of induced mood was also not statistically significant (*F*(4, 129) = 0.61, *p* = .658). However, the main effect of time (*F*(1.97, 254.38) = 7.44, *p* < .001, *η*_*p*_^*2*^ = .054) and the interaction between induced mood and time were significant (*F*(7.89, 254.38) = 2.01, *p* = .45, *η*_*p*_^*2*^ = .059). Following the significant interaction, we examined the simple main effect of time for each one of the induced moods. The simple main effect of time was not significant for the Positive (*F*(2, 48) = 0.21, *p* = .813), Self-Neutral (*F*(2, 50) = 1.38, *p* = .261) and Soundscape-Neutral conditions (*F*(2, 58) = 0.88, *p* = .421). On the other hand, the simple main effect of time was significant for the Negative (*F*(2, 54) = 5.84, *p* = .005, *η*_*p*_^*2*^ = .178) and Neutral conditions (*F*(2, 48) = 5.56, *p* < .01, *η*_*p*_^*2*^ = .19). Results for the pairwise comparisons of VAS Sadness are shown in Table 4 (Sadness).

*PANAS scores*. For the PANAS Positive affect (Fig 5), the main effect of induced mood was not found to be significant (*F*(4, 129) = 0.51, *p* = .731). However, the main effect of time (*F*(2, 258) = 28.40, *p* < .001, *η*_*p*_^*2*^ = .180) and the interaction between induced mood and time were significant (*F*(8, 258) = 3.65, *p* < .001, *η*_*p*_^*2*^ = .102). Following the significant interaction, we examined the simple main effect of time for each one of the induced moods. The simple main effect of time was not found to be significant for the Positive condition (*F*(2, 48) = 0.89, *p* = .416). However, the simple main effect of time was found to be significant for the Negative (*F*(2, 54) = 12.73, *p* < .001, *η*_*p*_^*2*^ = .320), Neutral (*F*(2, 48) = 12.21, *p* < .001, *η*_*p*_^*2*^ = .337), Self-Neutral (*F*(2, 50) = 9.51, *p* < .001, *η*_*p*_^*2*^ = .276) and Soundscape-Neutral conditions (*F*(1.60, 46.35) = 5.90, *p* = .009, *η*_*p*_^*2*^ = .169). Results for the pairwise comparisons of PANAS Positive affect are shown in Table 5 (Positive).

For the PANAS Negative affect (Fig 6), the main effect of induced mood (*F*(4, 129) = 1.46, *p* = .218) and the main effect of time were not found to be significant (*F*(1.90, 245.38) = 0.13, *p* = .871). However, the interaction was significant (*F*(7.61, 245.38) = 4.33, *p* < .001, *η*_*p*_^*2*^ = .118). Following the significant interaction, we examined the simple main effect of time for each one of the induced moods. The simple main effect of time was significant for the Positive (*F*(1.59, 38.14) = 8.34, *p* = .002, *η*_*p*_^*2*^ = .258), Negative (*F*(1.47, 39.76) = 5.46, *p* = .014, *η*_*p*_^*2*^ = .168) and Soundscape-Neutral conditions (*F*(1.61, 46.75) = 6.22, *p* = .007, *η*_*p*_^*2*^ = .177). On the other hand, it was not significant for the Neutral (*F*(1.42, 34.03) = 0.18, *p* = .760) and Self-Neutral conditions (*F*(2, 50) = 0.65, *p* = .528). Results for the pairwise comparisons of PANAS Negative affect are shown in Table 5 (Negative).

*PANAS-X scores*. For the PANAS-X Joviality (Fig 7), the main effect of induced mood was not significant (*F*(4, 129) = 1.09, *p* = .364). However, the main effect of time was significant (*F*(1.92, 247.39) = 25.30, *p* < .001, *η*_*p*_^*2*^ = .164), and the interaction between induced mood and time was also significant (*F*(7.67, 247.39) = 5.87, *p* < .001, *η*_*p*_^*2*^ = .154). Following the significant interaction, we examined the simple main effect of time for each one of the induced moods. The simple main effect of time was significant for all conditions: Positive (*F*(2, 48) = 4.06, *p* = .023, *η*_*p*_^*2*^ = .145), Negative (*F*(2, 54) = 18.86, *p* < .001, *η*_*p*_^*2*^ = .411), Neutral (*F*(2, 48) = 14.85, *p* < .001, *η*_*p*_^*2*^ = .382), Self-Neutral (*F*(2, 50) = 6.95, *p* = .002, *η*_*p*_^*2*^ = .218) and Soundscape-Neutral (*F*(1.53, 44.39) = 4.09, *p* = .033, *η*_*p*_^*2*^ = .124). Results for the pairwise comparisons of PANAS-X Joviality are shown in Table 5 (Joviality).

For the PANAS-X Sadness (Fig 8), the main effect of induced mood was not significant (*F*(4, 129) = 1.89, *p* = .116). However, the main effect of time (*F*(1.99, 256.45) = 27.90, *p* < .001, *η*_*p*_^*2*^ = .178) and the interaction between induced mood and time was significant (*F*(7.95, 256.45) = 5.07, *p* < .001, *η*_*p*_^*2*^ = .136). Following the significant interaction, we examined the simple main effect for each one of the induced moods. The simple main effect of time was significant for the Positive (*F*(1.51, 36.34) = 17.58, *p* < .001, *η*_*p*_^*2*^ = .423), Negative (*F*(2, 54) = 17.11, *p* < .001, *η*_*p*_^*2*^ = .388), Neutral (*F*(2, 48) = 6.29, *p* = .004, *η*_*p*_^*2*^ = .208), Self-Neutral conditions (*F*(2, 50) = 5.84, *p* = .005, *η*_*p*_^*2*^ = .189) but not the Soundscape-Neutral J-MIT (*F*(2, 58) = 1.24, *p* = .296). Results for the pairwise comparisons of PANAS-X Sadness are shown in Table 5 (Sadness).

*Gender differences*. To investigate the effect of gender, we conducted a three-way mixed-design ANOVA on time, induced mood, and gender for PANAS Positive affect and Negative affect. For the PANAS Positive affect, a significant main effect was observed for time (*F*(1.84, 141.92) = 8.72, *p* < .001, *η*_*p*_*^2^* = .102), but no significant main effects were found for induced mood (*F*(2, 77) = 0.55, *p* = .579, *η*_*p*_*^2^* = .014) or gender (*F*(1, 77) = 0.77, *p* = .384, *η*_*p*_*^2^* = .010). Moreover, the second-order interaction between time, induced mood, and gender was not significant (F(3.69, 141.92) = 0.38, *p* = .810). Concerning the first-order interactions, neither the interaction between time and gender (*F*(1.84, 141.92) = 1.61, *p* = .206) nor the interaction between induced mood and gender were found to be significant (*F*(2, 77) = 0.13, *p* = .875). The interaction between time and condition was significant (*F*(3.69, 141.92) = 5.44, *p* = .001, *η*_*p*_*^2^* = .124).

For PANAS Negative affect, no significant main effect was observed for time (*F*(2, 77) = 1.68, *p* = .189), induced mood (F(2, 77) = 2.25, p = .113), or gender (F(1, 77) = 1.59, p = .211). Moreover, the second-order interaction between time, induced mood, and gender was not significant (*F*(4, 77) = 0.72, p = .583). Concerning the first-order interactions, neither the interaction between time and gender (*F*(2, 77) = 0.67, *p* = .511) nor the interaction between induced mood and gender were found to be significant (*F*(2, 77) = 0.64, *p* = .532). The interaction between time and condition was significant (*F*(4, 77) = 9.24, *p* < .001, *η*_*p*_*^2^* = .194).

#### ToL task

**Fig 9:**
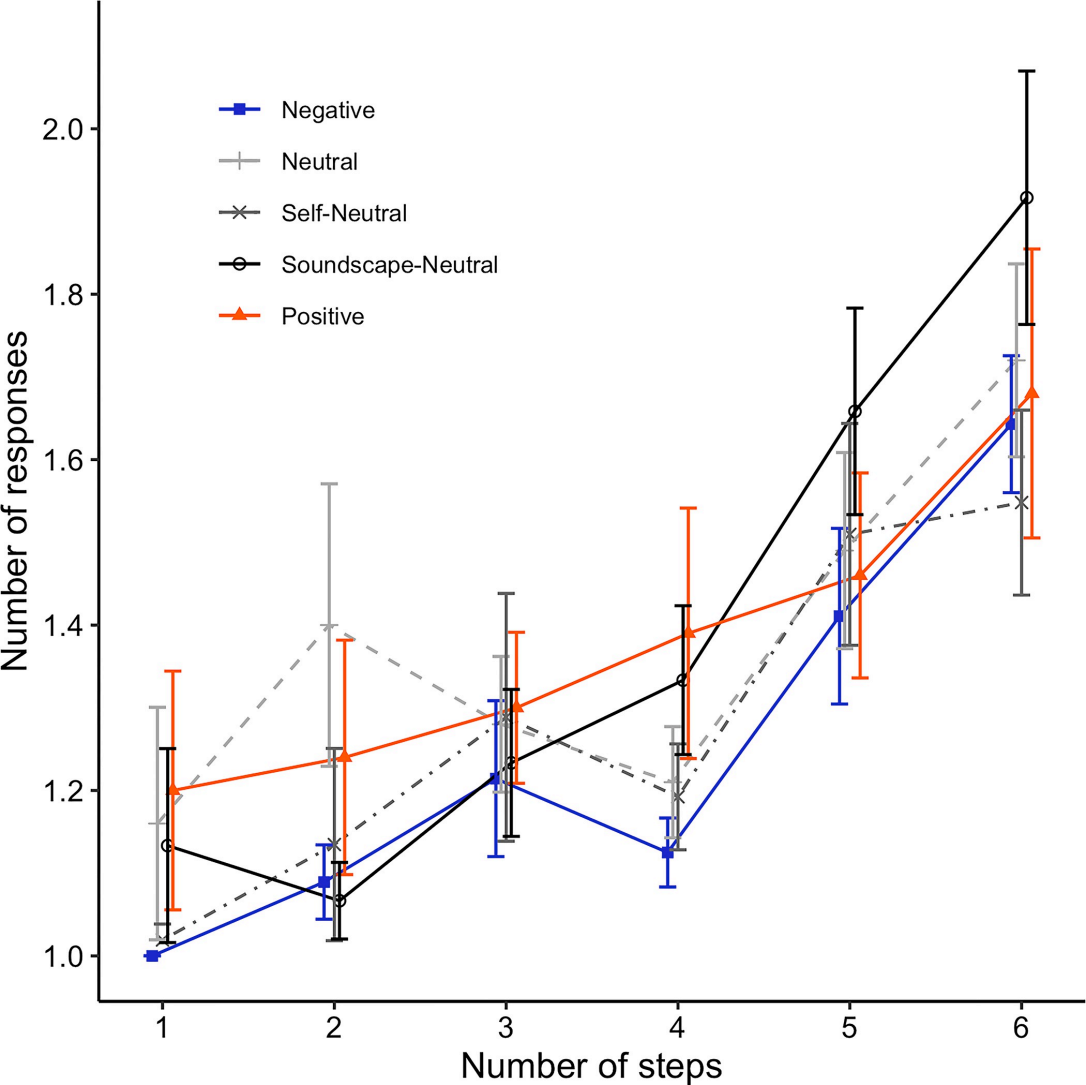
Mean numbers of button-presses in Tower of London trials for different levels of task difficulty. Each line represents a different condition. Error bars represent the standard error of the mean (SEM).

Fig 9 shows the mean number of button-presses given at every ToL trial until the correct answer was provided, for each level of task difficulty. We performed a mixed-design two-way ANOVA with difficulty (1 to 6) as the within-subjects factor and induced mood (Negative, Neutral, Self-Neutral, Soundscape-Neutral, Positive) as the between-subjects factor. Mauchly’s test indicated that the assumption of sphericity was violated (*χ*^2^(14) = 64.98, *p* < .001), therefore, the Greenhouse-Geisser correction was used (*ε* = 0.852). Even though the main effect of difficulty was found to be significant (*F*(4.26, 549.31) = 35.7, *p* < .001, *η*_*p*_^*2*^ = .217), there was no main effect of induced mood (*F*(4, 129) = .69, *p* = .603). Moreover, the interaction between both factors was not significant (*F*(17.03, 549.31) = 1.19, *p* = .265).

### Discussion

In Study 2, we assessed the effectiveness of an online implementation of the J-MIT in experimentally inducing mood states. Together with subjective ratings, we employed the ToL task to assess the potential effects of mood may have in executive control functions. Subjective ratings were collected at various timepoints to assess the duration of mood induction effects.

#### Immediate mood effects

Our primary interest was to verify whether the J-MIT was able to modulate participants’ mood in the intended direction (immediate effects) and whether such effects were still detectable after completion of the ToL task (sustained effects). Immediate mood effects were examined by inspecting the differences between the ratings given at baseline (T1) with ratings given immediately after completion of the J-MIT (T2). The constellation of results associated with the positive scales (VAS happiness, PANAS Positive affect, PANAS-X Joviality) and negative scales (VAS Sadness, PANAS Negative affect, PANAS-X Sadness) confirmed the effectiveness of the J-MIT on the immediate term. First, in the Positive condition, PANAS-X Joviality in positive scales increased significantly after induction; at the same time, negative scales (PANAS Negative affect and PANAS-X Sadness) significantly decreased after induction (Table 5). In sum, the directions of the changes were congruent with the target mood state (positive), suggesting that in the immediate term the positive mood induction was successful.

Next, in the Negative condition, all three positive scales significantly decreased after induction (i.e., VAS Happiness, PANAS Positive, PANAS-X Joviality). Likewise, all negative scales increased after induction (i.e., VAS Sadness, PANAS Negative, PANAS-X Sadness) (Tables 4 and 5). The directions of the changes were also congruent with the target mood state (negative), suggesting that, in the immediate term, the negative induction was also successful.

In the three neutral conditions (Neutral, Self-Neutral, Soundscape-Neutral), all positive scales consistently decreased after the mood induction. However, there were notable differences regarding the negative scales. In the Neutral condition, ratings given to the VAS Sadness significantly increased after induction (Table 4), while in the Self-Neutral conditions no changes were detected regarding the negative scales. On the other hand, in the Soundscape-Neutral condition the PANAS Negative decreased significantly following induction (Table 5). This suggests that relative to the Neutral and Self-Neutral conditions, the Soundscape-Neutral condition was more effective in attenuating both positive and negative mood in our participants.

What could be the possible cause for such difference? One explanation would be that the music stimulus employed in the Neutral and Self-Neutral conditions (“The Planets, Op. 32: VII. Neptune, the Mystic” by Holst) was perceived as having a negative valence by the participants. After the first data collection, participants were inquired via a questionnaire about what they thought was the most influential cause for whatever changes in mood they had experienced whether the sentences displayed on the screen, the music stimulus, the memory recall, or the display color. Remarkably, the most frequent answer was the music stimulus played during the induction procedure (51.4%), consistent with other studies (e.g., [43]). Based on that finding, in the second data collection, we replaced the auditory stimulus for soundscapes which are arguably much more emotionally neutral than complex music stimuli. Furthermore, in the Soundscape-Neutral condition, participants were given the opportunity to choose the auditory stimulus to be played during the study, making it in that respect more aligned with the Positive and Negative conditions.

#### Sustained mood effects and performance in the ToL task

Evidence for sustained effects following induction via the J-MIT was much less clear (Tables 4 and 5, T3-T1). In the Positive condition, though the PANAS-X Sadness continued to be lower at T3 relative to T1, there were no signs of remaining enhancements regarding the PANAS-X Joviality scores. For the Negative condition, even though the VAS Happiness remained significantly lower than baseline at T3 (Table 4) and the PANAS-X Sadness levels at T3 became significantly lower than baseline (Table 5), the increases in VAS Sadness and PANAS-Negative observed at T2 were no longer at T3. In the Neutral condition, three positive scales (VAS Happiness, PANAS Positive, PANAS-X Joviality) indicated continued decreases at T3. In the Self-Neutral condition, only the decrease observed in the VAS Happiness at T2 remained significant at T3. Finally, for the Soundscape-Neutral condition, only the scores of VAS Happiness remained smaller than baseline at T3.

We were not able to replicate previous results that indicated that positive and negative mood distinctively impair performance in the ToL task [8, 9]. Even though there was a main effect of task difficulty, we failed to detect a main effect of induced mood or an interaction between induced mood and task difficulty, indicating that participants made more errors as task difficulty increased but irrespective of the mood that was induced. One possible explanation for the lack of mood specific effects on ToL performance following the J-MIT are discrepancies in the number of trials and the overall duration of the ToL task across studies. Although such data were not reported in [8], in [9] the ToL task only employed three trials of high-difficulty levels. As such the overall duration of the ToL becomes much shorter, making the task more liable to display effects of induced mood.

#### Gender differences on mood ratings

Study 2 did not observe any significant gender effect or interactions for both PANAS Positive and Negative affect, deviating from the patterns observed in previous research. The potential influences from sample size, cultural background, and other variables present challenges in making direct comparisons with other studies [44, 45]. Nonetheless, within these constraints, our findings did not reveal any gender differences in our experimental design.

## General discussion

In this study, we conducted an online experiment to assess the effectiveness of the mood induction task [21] using a cohort of Japanese university students. In Study 1, we examined the valence communicated by Japanese translations of the Velten sentences and a newly constructed set of 60 self-referential neutral sentences. In Study 2, the sentences were embedded in an online implementation of the mood induction task and effects on mood were investigated using self-report ratings, and in a less overt manner using the ToL task. Results indicated that there were significant effects immediately following induction (i.e., immediate mood effects) and the directions of the changes in Positive, Negative, and Soundscape-Neutral conditions were mostly consistent with the intended target mood state. However, sustained mood effects were much less clear and we were not able to detect impaired ToL performance associated with positive and negative mood relative to neutral mood, as reported in [8]. Overall, these results suggest that the mood effects induced the online implementation of the J-MIT were not sufficiently strong to affect the performance of the subsequent ToL task, even though such effects were present immediately after induction. How induced mood effects decline over time is a commonly overlooked aspect in mood induction studies; a much more comprehensive and systematic understanding of the temporal dynamics underlying changes in mood in the laboratory and in daily life is necessary to determine when mood induction protocols can be expected to be effective in elucidating the mechanisms underpinning affective processes and how they can affect other cognitive functions.

The positive induction was successful immediately after and possibly during the J-MIT, given that the PANAS-X Joviality significantly increased after induction. However, similar effects were not observed in the other positive scales. One possibility would be that there was a non-negligible positive bias in the responses at the onset of the study [11]. The midpoint in the VAS is 50 by definition but for the participants in the Positive condition, the mean score of VAS Happiness at T1 was 62.1 (*SD* = 21.3) which was significantly larger than 50 (*t*(24) = 2.84, *p* < .01). This suggests that ratings using positive scales were already initially biased towards higher values at baseline; this would make more difficult to detect statistically significant effects following positive induction. Another possibility would be that the overall positive mood induction effects were smaller because of the online nature of this study. Inducing positive mood online is arguably challenging [46]. Despite that, the significant increase in PANAS-X Joviality suggests that participants in the Positive condition successfully experienced enhanced positive mood right after the J-MIT.

Among the three different neutral conditions tested in this study, the Soundscape-Neutral condition generated results that were most clearly distinguishable from the Positive and Negative conditions. The two other neutral conditions employed classical music pieces as the auditory stimulus. Mood effects induced by music stimuli are likely strongly modulated by individual preferences and cultural backgrounds. As such, unless tested in advance, we believe their use in mood induction protocols must always be considered with great caution. The neutral condition plays a crucial role as a comparison baseline against which the magnitude of effects induced by positive and negative valences are assessed. The current results confirmed that the Soundscape-Neutral is an appropriate neutral condition in the context of the J-MIT.

The results of the Neutral condition, as proposed in the original mood induction task [21], were similar to the Negative condition. One possibility was that Neutral condition incidentally induced a mood that was slightly more negative, increasing the similarity of the ratings given by participants in the Negative condition. Another possibility would be that the mood induced by the Negative condition was not sufficiently negative. One possibility would be that participants in the Negative condition may have inadvertently suppressed the arise of negative affect responses during induction. Emotional regulation mechanisms are adaptive, and the ability to control negative affective responses is often thought to be the hallmark of an optimally functioning individual [47]. For instance, a fMRI study that employed a mood induction protocol using emotional pictures found that activity in the visual cortex was lower during the induction of negative mood compared to positive mood [48]. The authors interpreted this result as a suppressive effect of an expected negative input. This aspect is only seldom considered in laboratory studies involving mood induction; future studies should attempt to systematically characterize individual factors that could be used to predict one’s susceptibility to mood induction effects. Notwithstanding, the current results indicate that all scales in the Negative condition significantly changed to towards the intended direction, much in contrast with the results from the Positive condition. This is consistent with previous findings showing that negative mood induction often leads to stronger effects when compared to positive mood induction [11].

Against our expectations, results from the ToL task did not show any mood-related modulatory effects. One possible explanation for this inconsistency is that the induced mood effects did not last the entire duration of the ToL task. In our online assessment, participants performed several trials of the same task difficulty to allow a more reliable measure. Though the average duration of the ToL task was not reported in [8, 9], it is likely that this difference made our manipulation, on average, it took 19 minutes for our participants to complete the ToL. Given that a study reported signs of decreased sadness 10 minutes after induction [25], this time lag can be viewed as relatively long. Another possible explanation would be that the feedback regarding the participants’ choices (correct choice/incorrect choice) inadvertently ended up affecting their mood states. While the exact number of trials utilized in [8] was not specified, our ToL task involved a greater number of high-difficulty trials compared to the procedure they referenced for the one-touch ToL task [49]. Consequently, it can be argued that the impact was potentially larger in our implementation of the ToL, since participants had to undergo more trials. This difference may have contributed, at least partially, to create the inconsistencies observed between both studies.

There are ways to address such problems in future studies. For example, a booster stimulus could be presented to help maintain the effects of the induced mood for prolonged periods of time, e.g., continuously exposing participants to the auditory stimuli employed during the mood induction phase throughout the entire experiment [1, 21]. Another approach would be to capitalize on the naturally occurring oscillations of mood in everyday life [6]. Monitoring participants mood states via experience sampling and inviting them to participate in laboratory studies when the reported mood state is consistent with the condition that needs to be tested could be a viable hybrid alternative that combines measurements both outside and inside the laboratory.

Finally, the experiments described here were conducted in September 2020 and February 2021. During that time, the COVID-19 pandemic imposed many restrictions in the lives of people around the world, and the situation was not different in Japan: University students had to stay off campus and most classes, if not all, were given online. Students were deprived from opportunities to socially interact with their peers in person, and in some cases, members of their families. It is possible that such restraints may have affected the baseline mood state of our participants. Therefore, it remains to be verified in future studies how generalizable the current results truly are.

## Supporting information

S1 File Used mood sentences. List of sentences employed in the study, with the mean “happiness” ratings and standard deviations collected in Study 1.
(XLSX)

S2 File Candidates of the neutral sentences. List of the candidate sentences for Self-Neutral, with the respective mean “happiness” ratings and standard deviations collected in a preliminary survey.
(XLSX)

S1 Text Preliminary research. Explanation about how the 60 Self-Neutral sentences were selected from the 100 candidate sentences. All candidate sentences are shown in the S2 File.
(DOCX)
