# Supplementary material for: Duration of mood effects following a Japanese version of the mood induction task
Source: PLoS One. 2024 Jan 5;19(1):e0293871. doi: 10.1371/journal.pone.0293871 (PMC10769078; doi:10.1371/journal.pone.0293871)
Supplement: S1 Text — Explanation about how the 60 Self-Neutral sentences were selected from the 100 candidate sentences. All candidate sentences are shown in the S2 File. (DOCX) [file pone.0293871.s003.docx]

# **S2 text**

## **Self-Neutral sentences**

The original Velten neutral statements describe facts about the US and the world that were pertinent to that time (e.g., “The machine dominated county posts for as long as anyone I could remember.”) or are passages devoid from context (e.g., “The desk was old, and scratched into its surface was a profusion of dates, initials, and pleading messages.”). The self-neutral sentences we employed for this study consisted of the 20 self-referential neutral sentences used in [26] plus 40 newly created sentences that were similar in style. First, we created a larger set of 80 candidate sentences that referred to ordinary everyday activities which are usually not associated with strong positive or negative emotions (e.g., “I sometimes watch the news”). We then recruited 104 undergraduate and graduate students (52 identified as women, 52 identified as men, mean age 21.7 years old, SD = 1.61, range 20-28 years old) using the services of Macromill, Inc. (Tokyo, Japan) to rate each one of the 80 candidate sentences plus the 20 sentences used in [26]. The instructions were as follows: “Please, read the statements below carefully. If you can relate with the described situation, try to imagine yourself in the shoes of someone in that situation, and rate your feelings using the following 9-point scale: 1: Extremely unhappy, 9: Extremely happy”. Ratings on this scale were used as a measure of the intensity of the positive or negative mood likely to be induced by each sentence; thus, sentences with average ratings in the middle range of the scale would be suitable to be included in a neutral condition. Data were collected in July 2020.

For each one of the sentences, a mean score of happiness was computed based on the collected ratings. From the 80 candidate sentences, we first selected those whose mean scores were within a half standard deviation from the mean score of the self-referential neutral sentences (4.78-6.04) used in [26] (which used the same 9-point scale of happiness). We then picked up the 40 sentences with the smallest variance. These 40 sentences were combined with the 20 self-referential neutral sentences used in [26] to form the set of 60 self-referential neutral sentences employed in this study. See S3 File for the full list of sentences with the respective mean ratings and SD.
